# Intensive Summer Intervention Drives Linear Growth of Reading Skill in Struggling Readers

**DOI:** 10.3389/fpsyg.2019.01900

**Published:** 2019-08-23

**Authors:** Patrick M. Donnelly, Elizabeth Huber, Jason D. Yeatman

**Affiliations:** ^1^Institute for Learning and Brain Sciences, University of Washington, Seattle, WA, United States; ^2^Department of Speech and Hearing Sciences, University of Washington, Seattle, WA, United States

**Keywords:** response to intervention, literacy, growth curves, dyslexia, summer intervention

## Abstract

A major achievement of reading research has been the development of effective intervention programs for struggling readers. Most intervention studies employ a pre-post design, to examine efficacy, but this precludes the study of growth curves over the course of the intervention program. Determining the time-course of improvement is essential for cost-effective, evidence-based decisions on the optimal intervention dosage. The goal of this study was to analyze reading growth curves during an intensive summer intervention program. A cohort of 31 children (6–12 years) with reading difficulties (*N* = 21 with dyslexia diagnosis) were enrolled in 160 h of intervention occurring over 8 weeks of summer vacation. We collected behavioral measures over 4 sessions assessing decoding, oral reading fluency, and comprehension. Mixed-effects modeling of longitudinal measurements revealed a linear dose-response relationship between hours of intervention and improvement in reading ability; there was significant linear growth on every measure of reading skill and none of the measures showed non-linear growth trajectories. Decoding skills showed substantial growth [*Cohen’s d* = 0.85 (WJ Basic Reading Skills)], with fluency and comprehension growing more gradually [*d* = 0.41 (WJ Reading Fluency)]. These results highlight the opportunity to improve reading skills over an intensive, short-term summer intervention program, and the linear dose-response relationship between duration and gains enables educators to set reading level goals and design a treatment plan to achieve them.

## Introduction

The most common learning disability in school-aged youth, developmental dyslexia, affects between 5 and 17% of children (Elliott and Grigorenko, 2014). Grounded in impaired decoding skill, dyslexia is characterized by disproportionate impairment in reading ability that cannot be explained by other contextual factors, such as poor reading instruction, or a major sensory deficit, such as poor visual acuity (Pennington, 2006; Shaywitz et al., 2008; Ferrer et al., 2010; Peterson and Pennington, 2012). Due to its high incidence, and the non-trivial impact on long-term academic achievement (Raskind et al., 1999; National Assessment of Educational Progress, 2006), a large body of scientific research has focused on the development of effective treatments for developmental dyslexia. Research starting in the 1980s has concluded that an effective intervention curriculum (a) explicitly teaches phonological awareness (Wagner and Torgesen, 1987; Ehri et al., 2001; Torgesen, 2006; Slavin et al., 2010; Suggate, 2010) (b) starts early at a child’s first indication of struggle (Torgesen, 1998; National Reading Panel, 2000; Shaywitz et al., 2008; Wanzek et al., 2013; Lovett et al., 2017), and (c) is multi-componential in nature layering in training in strategy, orthography, morphology and fluency (Lovett et al., 2008; Suggate, 2010; Wanzek and Vaughn, 2010; Morris et al., 2012; Stevens et al., 2016).

Beyond the efficacy of different curricula, important questions are left unanswered by previous research. Although the importance of intensive, early intervention is clear (Wanzek and Vaughn, 2007; Lovett et al., 2017), the reality remains that access to effective intervention is both a financial and emotional burden for families of struggling readers, especially during the school year (Delany, 2017). As such, many educators and advocates for struggling readers turn to intensive intervention programs during the summer break in attempt to close the gap between struggling children and their typical reading peers (Kristen et al., 2018). Past research has shown the benefit of summer reading programs that provide books to families and programing for oral reading and comprehension (Kim, 2006); moreover, a recent study shows that a highly intensive, summer intervention can effectively avoid the “summer slump” (Christodoulou et al., 2017). Growth curve analyses across the initial years of literacy development reveal lags during these critical transition periods of summer (Skibbe et al., 2012), but, to our knowledge, no studies have used similar techniques to examine growth curves during an intensive intervention implemented in the summer months. The goal of the present study is to characterize intervention-driven growth trajectories over the course of an intensive (4 h a day, 5 days a week) summer intervention program. In doing so, we can characterize intervention-driven learning trajectories over an isolated period of time without the confounding influence of concurrent educational activities and inform cost-effective decision-making on the appropriate duration of a summer intervention.

To date, the dominant model for studying intervention efficacy has been a pre-post design: children are tested before starting a program and after completing the program. The pre-post design has been used to determine not only those interventions that are more effective, but also has established foundational concepts about the mechanisms of dyslexia (Bradley and Bryant, 1983). Although some studies have argued that multiple measurements aren’t helpful in characterizing an individual’s level of disability (Schatschneider et al., 2008), multiple measurements are essential for making inferences about the time-course of learning over the course of an intervention (Verhoeven and van Leeuwe, 2009). Here, we use dense longitudinal measurements over the course of an intensive intervention to address two research questions: (1) What is the time course of learning for struggling readers enrolled in 8 weeks of intensive, individualized intervention? And (2) Is intervention growth most parsimoniously characterized by a linear model, or are there diminishing/accelerating returns with increasing hours of intervention? We employ the Lindamood-Bell *Seeing Stars* intervention because it was designed to be delivered in an intensive, one-on-one setting, over the course of the summer. In tandem with previous research looking at this program (Krafnick et al., 2011; Romeo et al., 2017), the goal of this work is not to compare the efficacy to other intervention approaches. Rather, we capitalize on the intensity of the program to model individual learning trajectories and understand the dose-response relationship between the number of treatment hours a child receives (dose) and their improvement in reading skills (response).

## Materials and Methods

### Participants

All parents of participants in the study provided written informed consent under a protocol that was approved by the UW Institutional Review Board and all procedures, including recruitment, child assent, and testing, were carried out under the stipulations of the UW Human Subjects Division. To ensure reproducibility of our findings, and to provide more detailed information on individual participant demographics, all the data and analysis code associated with this study is publicly available in an online repository^1^. Recruitment was based on parent report of reading difficulties and/or a clinical diagnosis of dyslexia. We intentionally recruited a diverse sample of struggling readers to study variability in response-to-intervention. A total of 31 children were enrolled in the intervention study, all of which were native English speakers with normal or corrected-to-normal vision. 21 participants reported a clinical diagnosis of dyslexia, and the other 10 participants reported struggles with reading but no formal diagnosis. Intervention participation occurred over the course of two summers (2016 and 2017), dividing the participants in two cohorts [Cohort 1 (*N* = 20), Cohort 2 (*N* = 11)]. Those enrolled also had no history of neurological damage or psychiatric disorder. Of those enrolled, 31 participants completed the entire study protocol (five total sessions): two baseline sessions and participation in the full 160 h of intervention (three additional sessions). Due to a scheduling issue, one participant received only 100 h. Growth estimates are based on data from the full sample (*n* = 31) given that the statistical technique used accounts for missing data. The sample consisted of 11 females (20 male), ranged in age from 7 to 13 (*M* = 9.4, SD = 1.7), and contained a heterogenous profile of reading ability centered 1.33 SD below the population average (*M* = 81.03, SD = 13.4), with IQ measures centered in the normal range (*M* = 101.4, SD = 10.6). Individual baselines (control period) were established in each child by conducting two experimental sessions prior to entry into the intervention program.

Sixteen children matched on age (*M* = 9.56, SD = 1.21), reading ability (*M* = 81.5, SD = 8.42), and IQ (*M* = 101.43, SD = 9.25) were recruited to participate as a control group. See Table 1 for descriptive statistics on both intervention and the control group. Data from 24 of the intervention participants and 16 of the 19 control participants has been previously published in a study exploring white matter plasticity during literacy intervention (Huber et al., 2018). The three control participants not included in this analysis were excluded as they did not fit the screening criteria of a diagnosis of dyslexia or parent report of reading impairment. Although there is some overlap in data, these analyses address the important goal of understanding the trajectory of behavioral growth [as opposed to white matter plasticity (Huber et al., 2018)]. The control group was used to model the effect of repeated testing and learning that would occur in a typical educational setting.

**TABLE 1:** Participant characteristics at intervention start (*n* = 47).

	**Intervention (*n* = 31)**	**Control (*n* = 16)**
***Characteristic (^∗^)***	***M (SD)***	***M (SD)***
Age (years)	9.4 (1.7)	9.56 (1.21)
Female (proportion)	0.35	0.38
WJ-IV Letter Word ID	76.4 (16)	76.63 (11.58)
WJ-IV Word Attack	88.6 (11.9)	89.56 (10.31)
WJ-IV Basic Reading Skills Composite	81 (13.4)	81.5 (8.42)
WJ-IV Oral Reading	79 (16.6)	75.94 (11.25)
WJ-IV Sentence Reading Fluency	75.5 (17.5)	76.13 (11.92)
WJ-IV Reading Fluency Composite	74 (18)	75.94 (11.25)
TOWRE-2 Sight Word Efficiency	74 (15.2)	70.75 (12.99)
TOWRE-2 Phonemic Decoding Efficiency	73.7 (12.4)	74.31(10.84)
TOWRE-2 TWRE Index Composite	72.6 (13.4)	71.06 (11.33)

*^∗^All scores are standard scores unless otherwise noted.*

Because our primary research questions focused on individual growth trajectories, and not on intervention efficacy, we did not use random assignment to intervention and control conditions (and, thus, we do not interpret our results as a randomized control trial). Instead, the control group was recruited in an independent period after the conclusion of the intervention periods. The control group underwent the same testing procedure but did not participate in the intervention. Testing sessions occurred during a “business-as-usual period,” during which the children attended their usual academic classes. Testing sessions were spaced equivalently to the intervention group with six participants completing all four sessions and the full sixteen completing at least two sessions. As the purpose of the control group was simply to (a) provide a comparison for repeated testing and change seen during typical schooling, and (b) to complement the individual baseline approach in examining changes outside of the intervention period, and not to prove/compare efficacy of a curriculum or pedagogical practice, an age and reading matched control group examined over this shorter period was appropriate. Furthermore, as the control group did not undergo an active comparison intervention, the results cannot be used to support the efficacy of any specific component of the intervention program.

### Experimental Sessions

**FIGURE 1:**
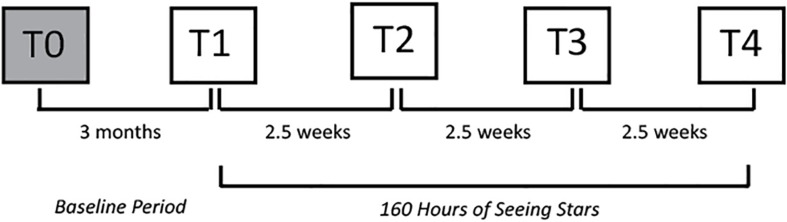
Schematic diagram of experimental design. Squares indicate experimental laboratory visits at the timepoints (T).

Each child participated in one baseline session 3 months prior to the beginning of the intervention program, a second baseline session immediately before starting the intervention, and three additional sessions to measure intervention-driven growth in reading skills. Except for the initial baseline session, each of the measurement sessions (including the second baseline) were spaced approximately 2.5 weeks apart over the course of the 8-week intervention, with midpoint measurements occurring during weeks 3 and 6. The second baseline session and the last measurement session immediately preceded/followed the intervention by, roughly, 2 weeks (see Figure 1).

Each experimental session included a comprehensive assessment of reading related skills administered by researchers at the University of Washington who were not involved in the administration of the intervention program and had no affiliation with Lindamood-Bell Learning Processes. The repeated battery for both baselines and the remaining visits included the Woodcock Johnson IV Test of Achievement (WJ) and the Test of Word Reading Efficiency-2 (TOWRE). From the WJ, the subtests administered were Letter-Word Identification (LWID), Word Attack (WA), Calculation (CALC), Oral Reading (OR), Sentence Reading Fluency (SRF), and Math Facts Fluency (MFF). These measures were combined to form the composite measures for Basic Reading Skills (BRS) (LWID + WA), Reading Fluency (RF) (OR + SRF), and Math Calculation Skills (MCS) (MFF + CALC). Composite measures were calculated using test-provided supplementary software. From the TOWRE, the subtests administered were the tests of sight-word and phonemic decoding efficiency (SWE and PDE), which formed the TWRE Index composite score, calculated based on the tables provided by the test. To account for test reliability with multiple measurements, alternative forms of the WJ and TOWRE battery were used on sequential visits. During the first baseline visit each child was also assessed for general cognitive abilities using the Weschler Abbreviated Scale of Intelligence – II (WASI), a composite of Vocabulary and Matrix Reasoning subtests).

### Reading Intervention

Two cohorts of participants were enrolled in 160 h of *Seeing Stars: Symbol Imagery for Fluency, Orthography, Sight Words, and Spelling* (Bell, 2007) over the course of 8 weeks of summer vacation. The first cohort was administered the intervention at three different *Lindamood-Bell Learning Centers* in the Seattle area in summer 2016, while the second cohort was administered the intervention at the Department of Speech and Hearing Sciences at the University of Washington. In both cohorts, the curriculum was administered by certified instructors from Lindamood-Bell. The *Seeing Stars* curriculum is a directed, individualized approach to training in phonological and orthographic processing skills. Employing a multi-sensory approach, the curriculum is incremental in training the foundations of literacy to systematically transition from letters and syllables, to words and connected texts. This concept of symbol imagery, with a well-documented research base (Kosslyn, 1976; Linden and Wittrock, 1981; Sadoski, 1983), is grounded in the idea that a robust understanding of letters and their associated sounds rests on the ability to recognize patterns and create mental representations at the level of the word. In a one-on-one setting with a certified instructor, children are encouraged to air-write the shape of letters and words, attend to their mouth movements, and visualize changes to words as the sounds are manipulated. In each lesson, an instructor will guide the student in a series of tasks that ask them to start with a word, visualize its constituent letters, link their related sounds, and develop a multisensory framework for approaching printed text. *Seeing Stars* presents a confluence of orthography, imagery, and meaning in providing directed instruction that extends from decoding and spelling skills to fluency and comprehension. Additional information can be found in other publications that have implemented this intervention (Krafnick et al., 2011; Christodoulou et al., 2017), and in the published intervention manual (Bell, 2013). The intervention was delivered at multiple locations and free of charge to all participants in this study.

### Statistics

All data analysis was done using MATLAB^®^ (MathWorks., 2017). Linear mixed effects models were used to analyze longitudinal change in reading skill as a function of hours of intervention. Mixed effects models can accommodate missing data, and participants do not have to be dropped due to missing data points. To determine which variables should be modeled as random effects, we started with the most parsimonious model (hours of intervention as a fixed effect and participant as a random effect) and then used Bayesian Information Criteria (BIC) and Akaike Information Criteria (AIC) to compare models with additional variables included as random effects. Following hierarchical pipeline, the following models were tested in determining the best-fitting model: (1) a single random intercept that varies by participant, (2) previous, with an independent random term for time grouped by participant, (3) previous, with an additional random intercept that varies by time, (4) previous, with a random-effects term for the intercept and time, and (5) previous, removing the intercept from the term added in model 4.

Following this approach for the each of the reading measures, we determined that the best fitting model included a subject specific random intercept and an independent random slope (model 2). To avoid over-fitting, we kept this same random effect structure and then added higher-order polynomial terms to the linear model to test for non-linear (quadratic) growth trajectories.

## Results

### Changes in Reading Skills During the Intervention

**FIGURE 2:**
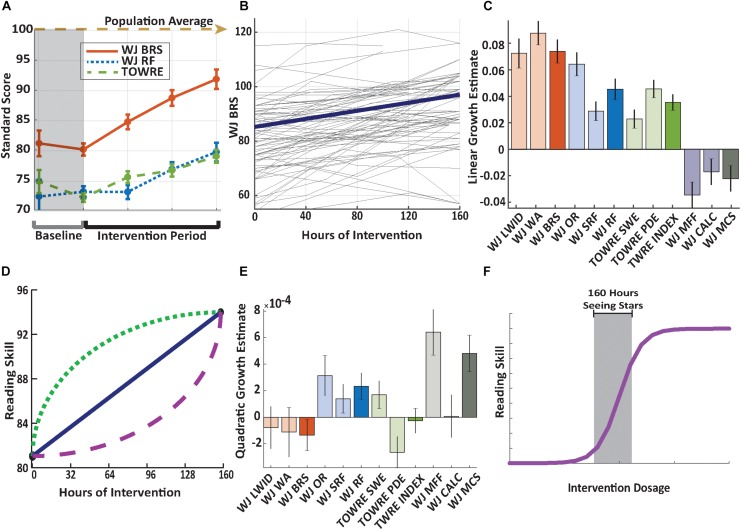
Significant growth across reading measures. **(A)** Mean growth of composite reading skills. Growth curves are plotted using the intercept and slope estimates from a linear mixed-effects model with session as a categorical variable. The dashed lines represent measurements during the baseline period. Results show growth across reading measures during the intervention period, and no change (or a decline) in scores during the baseline period. Error bars represent ± 1 SEM across participants. **(B)** Longitudinal growth of basic reading skills. Basic reading skills, measured by the Woodcock Johnson IV Basic Reading composite standard score, plotted for each individual child as a function of hours in the intervention. Participants completed up to 160 h of intervention. The bold line represents the linear fit based on a linear mixed-effects model (*p* = 3.53 × 10^–13^). **(C)** Growth rates across reading measures. Bar heights depict growth in skills per hour of intervention estimated based on a linear mixed-effects model. Error bars depict standard errors from the linear mixed-effects model. **(D)** Hypothetical growth trajectories. When reading skills are measured at multiple time-points over the course of an intervention, we might observe different patterns of growth that would be detected by adding quadratic terms to the model. **(E)** Comparison of a non-linear model of reading growth trajectories. Coefficients for the quadratic effects with error bars representing ± 1 SEM across participants. These effects were not significant for any of the reading measures, confirming that growth is predominantly linear. **(F)** A hypothetical dose-response curve. Our findings of linear growth in reading skills, without any significant deviations from linearity, indicate that 160 h of *Seeing Stars* lives in the shaded gray area of the dose response curve. Code and data to reproduce each figure is available in the online repository (e.g., https://github.com/yeatmanlab/growthcurves_public/blob/master/figure2.m for code to reproduce the figure). The tests abbreviated include the Woodcock-Johnson IV Tests of Achievement (WJ) Letter-Word Identification (WJ LWID), Word Attack (WJ WA), Basic Reading Skills composite (WJ BRS), Oral Reading (WJ OR), Sentence Reading Fluency (WJ SRF), Reading Fluency composite (WJ RF), Math Facts Fluency (WJ MFF), Calculation (WJ CALC), Math Calculation Skills composite (WJ MCS), Test of Word Reading Efficiency (TOWRE) Sight Word Efficiency (TOWRE SWE), Phonemic Decoding Efficiency (TOWRE PDE), and composite index (TWRE INDEX).

Longitudinal measurements of reading skills were conducted for 31 children who participated in an intensive summer reading intervention program (Lindamood-Bell *Seeing Stars*, see section “Materials and Methods”). The intervention involved one-on-one instruction, 4 h per day, 5 days per week, for eight weeks (160 h total). The study involved five experimental sessions: ∼3 months prior to the beginning of the intervention (*M* = 2.9, SD = 1 month), immediately before beginning intervention (8 days ± 6), after 48.5 (SD = 8.9) hours of instruction, 102.5(SD = 12.6) hours of instruction, and after completing the 160 h of intervention. Age-normed, standardized measures of reading skills were stable or declining, during the baseline period before the beginning of the intervention, and then increased systematically over the course of the intervention (Figure 2A).

The stability during the baseline period indicates that growth during the intervention period reflected the effect of participating in the intervention, rather than the effect of repeated measures or the passage of time (individual baseline). Specifically, WJ Basic Reading Skills (BRS) and RF measures were stable (BRS, *t*(30) = −0.89, *p* = 0.38; RF, *t*(30) = 0.68, *p* = 0.50), while standard scores on timed measures of decoding declined during the baseline period (TOWRE, *t*(30) = −3.55, *p* = 0.001). Table 2 reports the mean test scores at each time point and the full dataset is available online^2^.

**TABLE 2:** Reading battery results across the four experimental sessions and initial intake session related to participation in 160 h of directed reading intervention through the *Seeing Stars* curriculum of *Lindamood-Bell Learning Processes*.

**Descriptive and model fit statistics**														

	**Intake Session**		**Session 1**		**Session 2**		**Session 3**		**Session 4**		**Linear mixed effects model**			
**Test name**	**Mean**	**SD**	**Mean**	**SD**	**Mean**	**SD**	**Mean**	**SD**	**Mean**	**SD**	**Slope**	**SE**	***p*-value**	**95% CI**
LWID	78.5	14.4	76.4	16.0	79.9	15.6	84.7	15.0	88.0	12.6	0.07	0.01	3.8 × 10^(–8)^	(0.05, 0.09)
WA	88.0	12.1	88.5	11.9	95.8	13.1	95.4	11.5	103.5	11.9	0.09	0.01	1.4 × 10^(–15)^	(0.07, 0.11)
BRS	82.0	12.4	81.0	13.4	85.9	12.8	89.1	12.6	93.8	11.2	0.08	0.01	3.5 × 10^(–13)^	(0.06, 0.1)
OR	79.3	15.3	79.0	16.6	80.4	14.2	84.2	13.0	88.5	12.0	0.06	0.01	1.3 × 10^(–9)^	(0.04, 0.08)
SRF	74.1	16.0	75.5	17.5	75.2	16.6	77.2	15.9	78.8	15.5	0.02	0.01	0.003	(0.01, 0.04)
RF	73.3	16.4	74.0	18.0	75.1	15.9	77.2	15.8	80.1	15.1	0.04	0.01	6.5 × 10^(–6)^	(0.02, 0.06)
SWE	75.2	13.5	74.0	15.2	74.0	16.1	76.5	14.5	78.2	16.4	0.02	0.01	0.003	(0.01, 0.04)
PDE	77.7	10.8	73.7	12.4	81.1	11.8	80.3	11.1	83.1	11.7	0.04	0.01	6.1 × 10^(–9)^	(0.03, 0.06)
TWRE	75.4	11.7	72.6	13.4	76.3	13.5	77.1	12.4	79.6	13.7	0.03	0.01	4.1 × 10^(–7)^	(0.02, 0.05)
MFF	86.0	16.5	84.5	17.2	81.0	15.8	77.5	17.5	81.0	15.5	–0.03	0.01	0.001	(−0.05, −0.01)
CALC	87.0	0.0	85.6	13.6	83.8	12.9	84.1	14.3	82.7	13.4	–0.02	0.01	0.118	(−0.04, 0)
MCS	77.0	0.0	84.3	14.9	81.2	14.0	79.3	14.7	80.5	13.1	–0.03	0.01	0.002	(−0.04, −0.01)

*Means and standard deviations (SD) are shown as well as the model statistics from the linear mixed effects model analysis. The model analysis includes the group slope estimate for rate of change in RTI (Slope), significance value (*P*-value), and confidence interval (95% CI). Reading battery consists of the Woodcock-Johnson IV Letter-Word Identification (LWID), Word Attack (WA), Basic Reading Skills (BRS), Oral Reading (OR), Sentence Reading Fluency (SRF), Reading Fluency (RF), Math Facts Fluency (MFF), Calculation (CALC), and Math Calculation Skills (MCS); Test of Word Reading Efficiency 2 Sight Word Efficiency (SWE), Phonemic Decoding Efficiency (PDE), and the TWRE Reading Index (TWRE). All scores presented are standardized measures.*

To compare the effects of the intensive intervention to changes that might be observed in a typical classroom setting (or due to repeated testing), we compared growth in the intervention participants to an age and reading skill matched control group that did not participate in the intervention. The control group did not show improvements in any of the age-normed composite reading measures across experimental sessions (Supplementary Figures S1, S2 and Supplementary Table S1), confirming that growth in reading scores is not due to repeated testing. Moreover, there was a significant group (intervention versus control) by time (days) interaction for all composite measures (*p*s < 0.025) confirming that the growth observed in the intervention group is significantly greater than the control group (see Supplementary Table S1).

To summarize growth for each measure, we fit a linear mixed effects model where changes in reading scores were modeled as a function of hours of intervention, with each individual’s slope and intercept included as independent random effects within the model (see sections “Materials and Methods” and “Statistics”). Figure 2B shows growth trajectories for each individual subject: Even though there was substantial variability in initial reading scores and age, there was steady growth during the intervention. All measures of reading skills showed significant intervention-driven growth (Figure 2C). To investigate the influence of participant heterogeneity in these results, we performed a correlational analysis of individual growth rates (linear fits to each reading composite measure) with age and initial reading score, as measured by the WJ BRS composite. Age was not predictive of the linear growth observed in in any composite measure, indicating that improvements were equivalent across the broad age range sampled here (WJ BRS, *r*(31) = 0.08, *p* = 0.67; WJ RF, *r*(31) = 0.12, *p* = 0.52; TWRE Index, *r*(31) = 0.05, *p* = 0.78). There was a significant negative relationship between initial BRS and growth rate, as indexed by WJ BRS and WJ RF, indicating that the intervention stimulated the greatest change in the subjects who began with the lowest initial reading scores [WJ BRS, *r*(31) = −0.58, *p* < 0.001; WJ RF, *r*(310 = −0.45, *p* = 0.01)]. A negative relationship was also observed between initial BRS and growth in the TWRE Index measure, but the effect was not significant [*r*(31) = −0.33, *p* = 0.06].

Using a mixed effects model to compare growth rates between the binned timed and untimed reading measures we found that untimed measures (Woodcock Johnson IV, Word Attack (WA), Letter-Word Identification (LWID)) showed more rapid growth than timed measures [TOWRE, Sight Word Efficiency (SWE), Phonemic Decoding Efficiency (PDE)] (β = 1.87, *t*(60) = 4.68, *p* = 1.67 × 10^–5^). The untimed measure of pseudo word decoding (WA) showed the greatest rate of growth. This pattern was paralleled for measures of RF, with the untimed task (OR) improving more than the timed comprehension task (SRF).

To control for familiarity with testing, and examine whether learning generalizes to other academic skills, we also collected measures of mathematical skill. We found that Woodcock Johnson Math Facts Fluency (MFF), a timed measure of arithmetic skill, and Calculation (CALC), an untimed measure of quantitative skill, were stable, or declining, over the intervention period (Figure 2C and Table 1). These results indicate that students’ growth in reading skills were due to the training program rather than due to the Hawthorne effect (Cook, 1962), where scores increase due to repeated testing. Table 1 details the results of the linear mixed-effects modeling analysis for each of the reading-related behavioral measures.

### Growth in Reading Skills Is Not Significantly Different Than Linear

The primary goal of this study was to determine the typical shape of the growth curve over the summer intervention program. In our sample, 160 h of *Seeing Stars* produces, on average a 0.7 SD increase in word reading scores (Table 1); however, this growth could occur during the first few weeks of intervention with reading skills remaining roughly constant after the beginning of the program. Alternatively, many hours of training may be required to affect any change in scores. Figure 2D illustrates hypothetical dose-response curves that achieve the same result but have vastly different implications for how we might optimize intervention practice. The dotted curve demonstrates a rapid initial response which saturates toward the end of the intervention. This growth curve would imply that there are diminishing returns for a longer intervention and could be detected based on a significant negative quadratic effect in the model. The dashed curve demonstrates slow initial response with an accelerating rate of growth after more hours of intervention. This growth curve would imply that a longer intervention is required to realize the benefits of the curriculum and could be detected based on a significant positive quadratic effect in the model. Finally, the solid curve demonstrates a constant linear rate of change from start to finish. This growth trajectory would imply that each hour of intervention provides a subsequent unit growth in reading skill.

To detect potential non-linearity in the dose-response relationship between hours of intervention and reading growth, we added a higher order polynomial term (quadratic) to the mixed effects model. These models included the same random effects structure (see section “Materials and Methods”) to avoid over-fitting the model. We used AIC and BIC to compare the goodness-of-fit for models that include (a) only a linear term and (b) linear and quadratic terms to determine if a more complex model, with a non-linear growth rate, would be a better fit to the data than the more parsimonious linear model.

Our results indicate that the more complex model is not a significantly better fit to the data than the linear model. Figure 2E shows the coefficients for the quadratic terms; no reading measure showed significant quadratic effects (Supplementary Figure S3 shows the same analyses for raw scores). Incorporating a cubic term also did not improve the model fit for any reading measure. Hence, we conclude that over 160 h of intervention, improvements in reading skills follow a predominantly linear trajectory.

To determine if a lack of statistical power was responsible for the null result, we conducted a similar mixed model analysis measuring change in reading score as a function of time (by session number), rather than hours of intervention, and included data from the initial baseline session (T0 in Figure 1) through the third intervention session (T3 in Figure 1). In this case, we know that growth is non-linear since reading scores were stable between T0 and T1 and then increased after T1. Thus, this analysis tests our ability to detect a quadratic effect given the measurement variability and sample size in our study. We find a significant quadratic effect on the WJ BRS composite (β_2_ = 1.04, *p* = 0.009) indicating the accelerating growth starting at the beginning of the intervention period and confirming that the linear dose-response relationship seen during the intervention was not the result of a lack of statistical power. Although this does not negate the possibility of a small non-linear effect given a larger sample, it does add support to the finding that growth is predominantly linear and that, within an individual, growth during the intervention period is significantly steeper than during the control period.

## Discussion

Enrollment in 160 h of intensive, one-on-one intervention over the course of the summer led to systematic linear growth in reading skills, including real and pseudo-word decoding, reading fluency, and comprehension. This contrasted with stability or decline seen during a pre-intervention baseline period (individual baseline), and lack of change seen in a group of age- and reading skill-matched control participants. Importantly, reading skills increased linearly with each hour of intervention, carrying practical implications for decision making around intervention policy and practice. However, since we have not directly compared this intervention approach against other approaches, we cannot infer which specific factors in the intervention program were most important for success (e.g., one-on-one training versus the specific curriculum).

The finding that growth is linear draws attention to the issue of extrapolation from our data range. We can assume that linear growth would not persist indefinitely, and that at some intervention dosage (above 160 h) participants will stop benefiting from more hours of intervention. Dose-response curves typically follow a sigmoidal shape (depicted in Figure 2F), where small dosages provide limited returns, but as the dosage increases, the effect grows until the curve saturates indicating that added dosage provides limited returns. Our results indicate that 160 h of *Seeing Stars* fell in this intermediate range of the curve, where growth is roughly linear (Grayed out section of Figure 2F) due to the lack of significance of higher-order polynomial terms in the model. Future research will be needed to determine the minimum hours needed to produce an improvement and the saturation point of the curve.

Unique to this intervention study is the intensity of instruction: 160 h of instruction over an 8-week period. To our knowledge, this intensity is unmatched in the intervention literature [for review of intervention studies see Al Otaiba and Fuchs (2002), Wanzek et al. (2013)], and provides a strong paradigm to characterize individual growth and response to intervention. In the context of intervention research that gained traction in the 1990s, the recommended dosage has usually been determined by logistical feasibility, coherence with ongoing schooling, and motivated by the goal of enhancing decoding skill with the hope that improved decoding will extend to fluency and comprehension (Lyon and Moats, 1997). As an individual child’s response is so variable, there is no golden-rule for intervention dosage (Shaywitz and Shaywitz, 1994); rather, the onus on educators is to discover the appropriate dosage that gives the struggling reader the necessary decoding foundation to, theoretically, jumpstart their ability to catch up to their peers in the realm of fluent comprehension (Lyon and Moats, 1997; Torgesen, 2006; Vadasy et al., 2008). Here we find significant growth in both decoding skill and reading fluency, and provide data demonstrating how much improvement we can expect based on each hour of one-on-one intervention. Future work can characterize the factors that explain individual differences in growth trajectories as there was variability among subjects.

The predominant method for studying intervention efficacy is a pre-post design, where a single measurement before and a single measurement after intervention are used to determine the average amount of change in reading skills. This method has been extremely effective in answering the question of whether a given intervention program is effective in improving reading skill, estimate effect sizes for intervention-driven improvement in reading skill, and comparing the efficacy of different intervention approaches. One question that has remained unanswered by this design concerns the dose-response relationship between the amount of intervention and growth in reading skills. By conducting multiple experimental sessions over the course of the intervention we can develop models that help us answer important questions about intervention “*dosage”*: How much intervention is appropriate for a child? How much is too little? What is the most cost-effective way to achieve the greatest gains? Is this intervention worth the investment, and what should be the recommended dose for a given child? Given the linear dose-response relationship, we can infer that 80 h of intervention would produce half the amount of growth in each reading measure. However, it is also important to keep in mind that we characterized the dose-response curves for one specific intervention program, with a relatively small, heterogenous sample of subjects, and without an active control condition. Therefore, it will be important to extend this methodology with a more controlled study design, to more diverse samples and other intervention programs. Follow up studies of this nature will enable researchers to compare dose-response curves of component reading skills, and associate those growth curves with specific intervention techniques.

Our study, like others that have applied growth curve analyses (Lovett et al., 1994; Lyon and Moats, 1997; Torgesen et al., 1999, 2001; Stage et al., 2003; Vadasy et al., 2008; Skibbe et al., 2012), represents an effort to create a systematic method for determining the optimal intervention dosage that can inform how families and school districts allocate resources to support struggling readers. By looking at growth as a function of the hours and type of intervention, models of individual growth curves provide a tool for making cost-effective, evidence-based decisions about remediation. As the benefits of phonologically based intervention saturate (Lyon and Moats, 1997), the challenge of continued intervention is to generalize this growth to gains in fluency and comprehension (Lovett et al., 1994; Torgesen, 2006; Skibbe et al., 2012). Based on longitudinal measurements, researchers and educators alike can monitor the gains across reading-related skills to determine for each individual child the type and dosage that maximizes return on investment. With this information, parents and educators can weigh the costs and benefits to make informed decisions about their child’s learning. Likewise, school districts and policymakers can use such information to save resources in providing requisite accommodations.

Unlike previous intervention research, the current study does not seek to determine the efficacy of a specific curriculum; rather, we aim to use intervention as a means to illustrate the time course of learning, lending to the ability of future research to identify the hallmarks that define an effective, gold-standard model for the individualized intervention of struggling readers. Although the approach used in this study provides information that is useful for policy around cost-effective, evidence-based intervention, these results also open additional questions. For example, the *Seeing Stars* program is a multi-componential intervention, which presents challenges to studying the discrete benefits of any specific technique. The study utilized a non-intervention control group, rather than an active control group, which is effective in supporting intervention-related growth, but precludes the ability to support efficacy of the specific intervention techniques employed.

Another limitation of this study was its reliance on standardized measures to characterize growth. In an exploratory, *post hoc* analysis using the raw scores, significant linear growth was seen for all measures, and significant quadratic growth was seen in the timed measure of phonemic decoding (see Supplementary Figure S3). As such, corollary analyses using raw scores that are able to pick up on subtleties in growth is a necessary component of future studies looking into growth curve analyses. Additionally, the use of more comprehensive questionnaires exploring the educational history of participants could provide useful information regarding the effect of prior experience on response to intervention. Finally, long-term follow up measurements are critical for determining the ability of any given short-term intervention program to provide enduring benefits for struggling readers. Reading research should continue to strive for models that consider a child’s unique intellectual profile and educational experience to optimize the intervention strategy for long-term success.

## Data Availability

All data and analysis code associated with this manuscript is publicly accessible at the following link: https://github.com/yeatmanlab/growthcurves_public.

## Ethics Statement

All parents of participants in this study provided written informed consent under a protocol that was approved by the UW Institutional Review Board and all procedures, including recruitment, child assent, and testing, were carried out under the stipulations of the UW Human Subjects Division.

## Author Contributions

All authors designed the study. PD collected the data. PD and JY analyzed the data and wrote the manuscript.

## Conflict of Interest Statement

The authors declare that the research was conducted in the absence of any commercial or financial relationships that could be construed as a potential conflict of interest.

## Footnotes

1 https://github.com/yeatmanlab/growthcurves_public

2 https://github.com/yeatmanlab/growthcurves_public/tree/master/data

## Supplementary Material

FIGURE S1 Control group comparison. This figure is identical in analysis to Figure 2A, for the cohort of 16 age and reading ability matched control participants. Each point represents the model fit curve using the linear effects model across control period sessions. Results show that the control participants experienced no significant growth of composite measures in response to participation as controls over the period of observation.

FIGURE S2 Growth statistics from linear mixed effects model. This figure is identical to Figure 2C in its analysis of the growth statistics from the linear mixed effects model, but for the cohort of 16 age- and reading ability-matched control participants. Results show that no measure demonstrated significant change, with growth trends in varied directions. Growth estimate axis is scaled in terms of standard score increase per unit day of control condition.

FIGURE S3 Exploratory analysis of growth estimates for raw scores. **(A)** This figure is identical to Figure 2C in its analysis of the growth statistics from the linear mixed effects model, but using the raw score measures, where available. Results mirror those for the standard scores with significant effects across all measures. Growth estimate axis is scaled in terms of raw score increase per unit day. **(B)** This figure is identical to Figure 2E in its analysis of quadratic growth estimates but using the raw score measures. Results mirror those for the standard measures for all but the TOWRE Phonemic Decoding (PDE) measure, which saw a statistically significant negative effect. These exploratory, *post-hoc* results are compelling and evidence the important consideration of raw scores in future studies.

TABLE S1 Reading battery results across the experimental sessions and initial intake session related to participation as a matched control. Descriptive measures including mean and standard deviation (SD) are shown as well as the model statistics from the linear mixed effects model analysis. The model analysis includes the group slope estimate for rate of change in RTI (Slope) and significance value (*P*-value). The interaction model analysis included all participants and lists the beta value (Coefficient) and significance (*P*-value) of the effect of Time (days) X Group [Intervention (*N* = 37) or Control (*N* = 16)]. Non-significant interaction coefficients seen in the SRF and SWE measures reveals high variability and added noise in the control group as a result of small sample size. Reading battery consists of the Woodcock-Johnson IV Tests of Achievement Letter-Word Identification (LWID), Word Attack (WA), Basic Reading Skills Composite (BRS), Oral Reading (OR), Sentence Reading Fluency (SRF), Reading Fluency Composite (RF), Math Facts Fluency (MFF), Calculation (CALC), and Math Calculation Skills Composite (MCS); Test of Word Reading Efficiency 2 Sight Word Efficiency (SWE), Phonemic Decoding Efficiency (PDE), and the TWRE Reading Index Composite (TWRE).
