# Supplementary figures and images for: Intensive Summer Intervention Drives Linear Growth of Reading Skill in Struggling Readers
Source: Front Psychol. 2019 Aug 23;10:1900. doi: 10.3389/fpsyg.2019.01900 (PMC6716466; doi:10.3389/fpsyg.2019.01900)

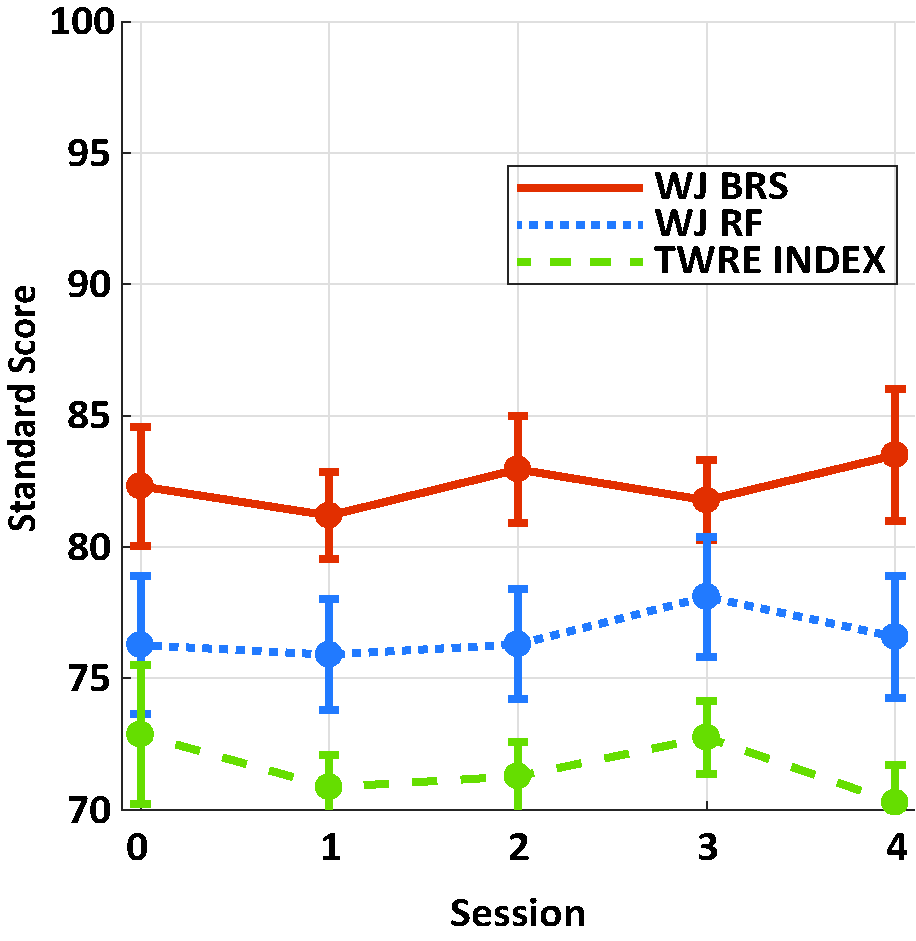

Supplement: FIGURE S1 — Control group comparison. This figure is identical in analysis to Figure 2A, for the cohort of 16 age and reading ability matched control participants. Each point represents the model fit curve using the linear effects model across control period sessions. Results show that the control participants experienced no significant growth of composite measures in response to participation as controls over the period of observation. [file Image_1.tif]

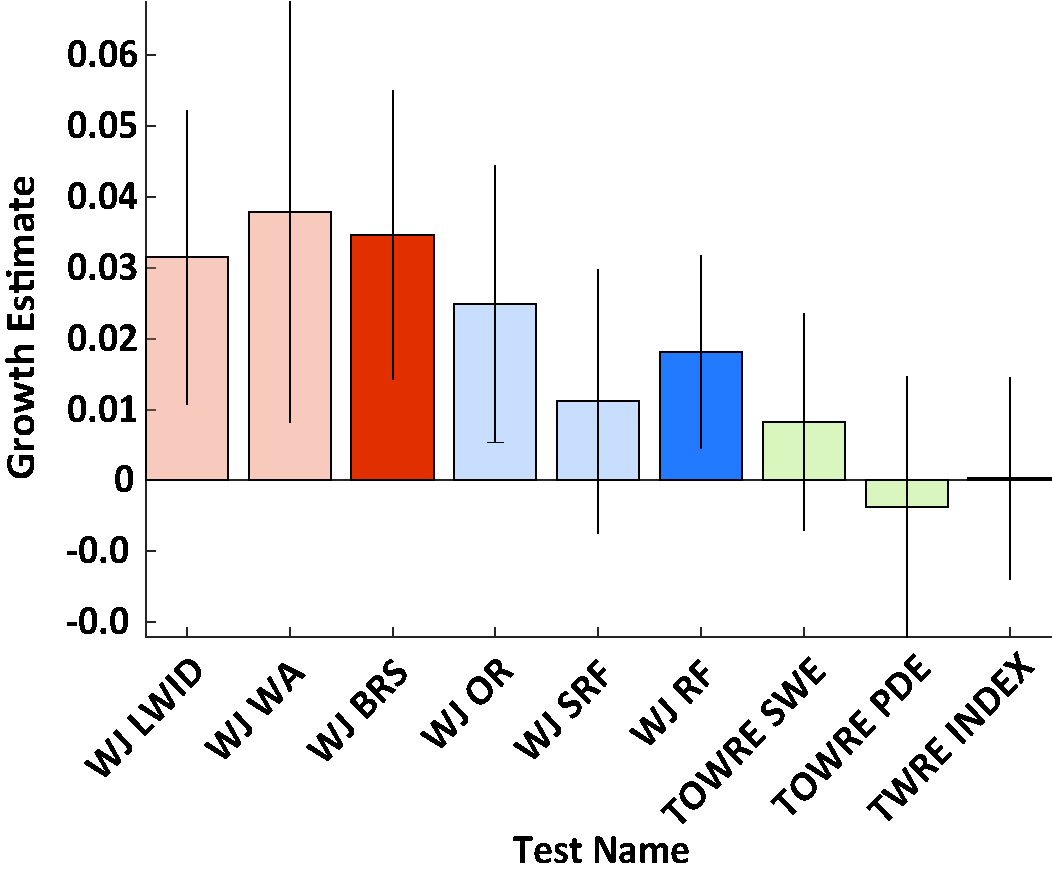

Supplement: FIGURE S2 — Growth statistics from linear mixed effects model. This figure is identical to Figure 2C in its analysis of the growth statistics from the linear mixed effects model, but for the cohort of 16 age- and reading ability-matched control participants. Results show that no measure demonstrated significant change, with growth trends in varied directions. Growth estimate axis is scaled in terms of standard score increase per unit day of control condition. [file Image_2.tif]

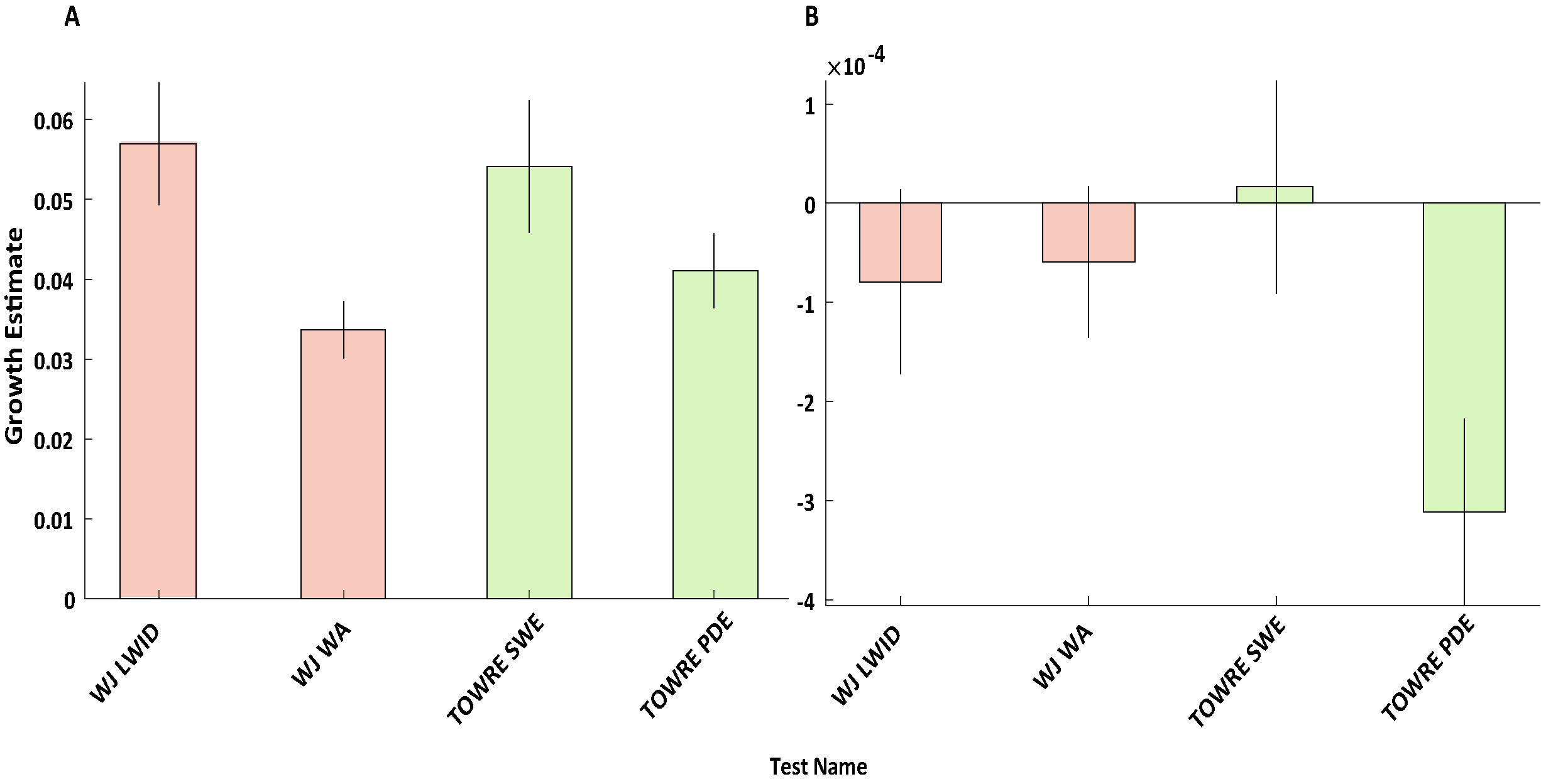

Supplement: FIGURE S3 — Exploratory analysis of growth estimates for raw scores. (A) This figure is identical to Figure 2C in its analysis of the growth statistics from the linear mixed effects model, but using the raw score measures, where available. Results mirror those for the standard scores with significant effects across all measures. Growth estimate axis is scaled in terms of raw score increase per unit day. (B) This figure is identical to Figure 2E in its analysis of quadratic growth estimates but using the raw score measures. Results mirror those for the standard measures for all but the TOWRE Phonemic Decoding (PDE) measure, which saw a statistically significant negative effect. These exploratory, post-hoc results are compelling and evidence the important consideration of raw scores in future studies. [file Image_3.tif]
