# Supplementary material for: Intensive Summer Intervention Drives Linear Growth of Reading Skill in Struggling Readers
Source: Front Psychol. 2019 Aug 23;10:1900. doi: 10.3389/fpsyg.2019.01900 (PMC6716466; doi:10.3389/fpsyg.2019.01900)
Supplement: TABLE S1 — Reading battery results across the experimental sessions and initial intake session related to participation as a matched control. Descriptive measures including mean and standard deviation (SD) are shown as well as the model statistics from the linear mixed effects model analysis. The model analysis includes the group slope estimate for rate of change in RTI (Slope) and significance value (P-value). The interaction model analysis included all participants and lists the beta value (Coefficient) and significance (P-value) of the effect of Time (days) X Group [Intervention (N = 37) or Control (N = 16)]. Non-significant interaction coefficients seen in the SRF and SWE measures reveals high variability and added noise in the control group as a result of small sample size. Reading battery consists of the Woodcock-Johnson IV Tests of Achievement Letter-Word Identification (LWID), Word Attack (WA), Basic Reading Skills Composite (BRS), Oral Reading (OR), Sentence Reading Fluency (SRF), Reading Fluency Composite (RF), Math Facts Fluency (MFF), Calculation (CALC), and Math Calculation Skills Composite (MCS); Test of Word Reading Efficiency 2 Sight Word Efficiency (SWE), Phonemic Decoding Efficiency (PDE), and the TWRE Reading Index Composite (TWRE). [file Table_1.docx]

**Supplementary Table 1:** Reading battery results across the experimental sessions and initial intake session related to participation as a matched control. Descriptive measures including mean and standard deviation (SD) are shown as well as the model statistics from the linear mixed effects model analysis. The model analysis includes the group slope estimate for rate of change in RTI (Slope) and significance value (P Value). The interaction model analysis included all participants and lists the beta value (Coefficient) and significance (P Value) of the effect of Time (days) X Group (Intervention (N=37) or Control (N=16)). Non-significant interaction coefficients seen in the SRF and SWE measures reveals high variability and added noise in the control group as a result of small sample size. Reading battery consists of the Woodcock-Johnson IV Tests of Achievement Letter-Word Identification (LWID), Word Attack (WA), Basic Reading Skills Composite (BRS), Oral Reading (OR), Sentence Reading Fluency (SRF), Reading Fluency Composite (RF), Math Facts Fluency (MFF), Calculation (CALC), and Math Calculation Skills Composite (MCS); Test of Word Reading Efficiency 2 Sight Word Efficiency (SWE), Phonemic Decoding Efficiency (PDE), and the TWRE Reading Index Composite (TWRE).
